# Evaluation of Krill Meal in Commercial Diets for Juvenile Swimming Crab (*Portunus trituberculatus*)

**DOI:** 10.1155/2022/3007674

**Published:** 2022-11-21

**Authors:** Chen Guo, Min Jin, Lefei Jiao, Shichao Xie, Xiangsheng Zhang, Jiaxiang Luo, Tingting Zhu, Qicun Zhou

**Affiliations:** Laboratory of Fish and Shellfish Nutrition, School of Marine Sciences, Ningbo University, Ningbo 315211, China

## Abstract

An 8-week feeding trial was carried out to assess the effect of dietary krill meal on growth performance and expression of genes related to TOR pathway and antioxidation of swimming crab (*Portunus trituberculatus*). Four experimental diets (45% crude protein and 9% crude lipid) were formulated to obtain different replacements of fish meal (FM) with krill meal (KM); FM was replaced with KM at 0% (KM0), 10% (KM10), 20% (KM20), and 30% (KM30); fluorine concentration in diets were analyzed to be 27.16, 94.06, 153.81, and 265.30 mg kg^−1^, respectively. Each diet was randomly divided into 3 replicates; ten swimming crabs were stocked in each replicate (initial weight, 5.62 ± 0.19 g). The results indicated that crabs fed with the KM10 diet had the highest final weight, percent weight gain (PWG), and specific growth rate (SGR) among all treatments (*P* < 0.05). Crabs fed with the KM0 diet had the lowest activities of total antioxidant capacity (T-AOC), total superoxide dismutase (SOD), glutathione (GSH), and hydroxyl radical scavenging activity and had the highest concentration of malondialdehyde (MDA) in the hemolymph and the hepatopancreas (*P* < 0.05). In the hepatopancreas, the highest content of 20:5n-3 (EPA) and the lowest content of 22:6n-3 (DHA) were shown in crabs fed with the KM30 diet among all treatments (*P* < 0.05). With the substitution level of FM with KM gradually increasing from 0% to 30%, the color of the hepatopancreas changed from pale white to red. Expression of *tor*, *akt*, *s6k1*, and *s6* in the hepatopancreas was significantly upregulated, while *4e-bp1*, *eif4e1a*, *eif4e2*, and *eif4e3* were downregulated with dietary replacement of FM with KM increasing from 0% to 30% (*P* < 0.05). Crabs fed with the KM20 diet had notably higher expression of *cat*, *gpx*, *cMnsod*, and *prx* than those fed with the KM0 diet (*P* < 0.05). Results demonstrated that 10% replacement of FM with KM can promote growth performance and antioxidant capacity and notably upregulate the mRNA levels of genes related to TOR pathway and antioxidant of swimming crab.

## 1. Introduction

Fish meal (FM) has been regarded as the main and important protein ingredient in aquafeeds due to its high protein, balanced essential amino acid profile, low carbohydrate, good palatability, less antinutritional factors, and easily digestible and absorbable by aquatic animals [1–3]. Nevertheless, with the overfishing of marine fishery resources, the abnormal weather caused by El Nino, and the rapid development of aquaculture in developing countries, especially in China and other Southeast Asian countries, the world's FM demand exceeds supply and the price skyrocketed [1]. According to statistics, an increasing proportion of food fish is now provided by aquaculture, and the production is growing at an average rate of 9% per year [4, 5]. At the same time, aquafeeds for FM demand is high, whose market accounted for about 60% of total FM [3]. Therefore, many studies focused on the exploitation of alternative protein sources of FM, such as plant protein sources and byproducts of terrestrial animal and marine fish [1]. Whereas, although protein sources and species have a great variety, the utilization of plant protein is restricted by the imbalance of amino acids, the existence of antinutritional factors, and poor palatability, especially for marine fish and crustaceans [1, 3, 6, 7]. Moreover, the byproducts of terrestrial animal and marine fish are more than the consumption of human beings, and they cannot become the primary protein source of aquafeeds due to the small number of resources and easy metamorphism [2]. Therefore, exploiting new protein sources with large resources is an urgent task for aquaculture industry.

Antarctic krill (*Euphausia superba*) is an important species of fisheries in the world, whose biomass is estimated to be between 342 and 356 million tons [3, 8]. Antarctic krill is regarded as an excellent protein source and a suitable replacement for FM due to its balanced amino acids and fatty acids [9]. Generally, the crude protein content of Antarctic KM ranges from 490 to 720 g kg^−1^, and the crude lipid content is 120-170 g kg^−1^ [10, 11]. Moreover, docosahexaenoic acid (DHA) and eicosapentaenoic acid (EPA) are rich in KM, and 30% to 65% of the total fatty acids were presented as the form of phosphoglyceride (PG), and the phospholipid form of n-3 fatty acids has been demonstrated to be more efficiently absorbed by aquatic animals [12, 13], whereas the conventional composition of Antarctic KM is also affected by different growth stages and processing methods [14]. Moreover, Antarctic KM is rich in carotenoids, especially astaxanthin (AX). AX is a natural antioxidant that has significant effect on scavenging free radicals, and its antioxidant power is over 500 times of vitamin E [15, 16]. Previous studies have evaluated the feasibility of dietary replacement of KM in fish and crustacean; the results indicated that dietary KM could promote growth performance and feed intake, improve the meat quality, and enhance immunity [3, 11, 17–19]. Nevertheless, some studies have also reported that high content of fluorine in KM might be a major influence factor to limit its utilization [10, 20]. Fluorine has mainly existed in the exoskeleton of krill. Therefore, removing the exoskeletons can be an effective way to reduce the fluorine level because fluorine could migrate from the exoskeleton into the muscle after the krill dies [21, 22].

Swimming crab (*Portunus trituberculatus*) is a familiar marine crustacean in China. Recently, it has become the most popular seafood due to its rich nutrition, delicious meat, and special taste [23]. By 2020, the total aquaculture production of swimming crab in China has reached 113,810 tons [24]. Several studies have evaluated the application of protein sources in swimming crab, such as soy protein concentrate and low-gossypol cottonseed protein concentrate [25, 26]. To our knowledge, there is no information on the use of KM as a substitute for FM in swimming crab. Hence, the present study purposed to evaluate the effects of dietary substitution of FM with KM on growth performance, body composition, hematological characteristics, amino acid composition, fatty acid profile, antioxidant capacity, and expression of genes related to TOR pathway.

## 2. Materials and Methods

### 2.1. Ethics Statement

The animal welfare and housing provisions of this study were in the Guide for the Use of Laboratory Animals of Ningbo University, and the experiment was approved by the Animal Research and Ethics Committee of Ningbo University [26].

### 2.2. Diet Preparation

The formulation and proximate compositions of the experimental diets are shown in Table 1. Four isonitrogenous (about 45.0% crude protein) and isolipidic diets (about 9.0% crude lipid) were formulated to satisfy the protein and lipid requirements for juvenile swimming crab [27]. The dietary FM was substituted by KM at 0% (KM0), 10% (KM10), 20% (KM20), and 30% (KM30), respectively. Each diet was supplemented with lysine and methionine for consistency. Tables 2 and 3 show the dietary amino acid profiles and fatty acid compositions in the present study. The process of feed production was referred to the previous study [27].

### 2.3. Feeding Trial and Rearing Conditions

Juvenile swimming crabs were all taken from fishery breeding bases (Ningbo, China). Domestication and grouping of swimming crabs could be referred to the previous study [26]. 120 swimming crabs with an initial weight of 5.62 ± 0.19 g were randomly selected and distributed into independent plastic baskets (35 cm × 30 cm × 35 cm) and were distributed into three cement pools (8.3 m × 3.5 m × 1.5 m). The internal distribution of the plastic basket is as follows: the inside of the basket is separated by a partition to provide a resting and feeding area for the experimental crabs, and a moderate amount of sand is added to the resting area. In addition, each plastic basket is embedded with a foam block of the right size, allowing it to float and facilitate feeding and observation during the experiment [28]. The feeding work was precisely arranged at 8 : 00 and 18 : 00 everyday. The number of crab's carapaces and remaining feed was recorded and removed before feeding. Approximately 20% of the seawater in the cement pool was replaced every day to control the seawater parameters as follows: water temperature: 28.9 to 31.0°C; salinity: 24.8 to 25.4 g L^−1^; pH: 7.7 to 8.0; ammonia nitrogen: under 0.05 mg L^−1^; and dissolved oxygen: not less than 6.0 mg L^−1^.

### 2.4. Sample Collection

After the 8-week feeding trial ended, the crabs were counted and weighed in each replicate. The hemolymph was extracted from the pericardial cavity of the swimming crab by using a 2 ml syringe. In addition, the same number of samples was ensured for each repetition and then loaded the hemolymph into the same centrifuge tube to ensure the consistency of the sampling. The hemolymph was allowed to stand at 4°C overnight to remove the gelatinous material, and the remaining supernatant was stored at -80°C for analysis. The hepatopancreas and the muscles were sampled quickly and frozen in liquid nitrogen for analysis. In addition, the hepatopancreas samples for enzyme activity analysis and gene expression were immediately added into 2 ml centrifuge tubes and 1.5 ml centrifuge tubes with RNA stabilization solution (Beijing Solarbio Science & Technology Co., Ltd.). Except for the sample containing RNA protection solution should be stored at -80°C after standing at 4°C overnight, the rest of the samples were immediately stored at -80°C after sample collection.

### 2.5. Proximate Composition Analysis

Proximate composition of diets and tissues was drawn on the method of the AOAC (Association of Official Analytical Chemists) [29]. Moisture was determined by continuous drying at 105°C to constant weight. The crude protein content was measured by the method of Dumas combustion, and the content of crude lipid was analyzed by the ether extraction method.

### 2.6. Antioxidant Capacity Assays

After the hepatopancreas samples were thawed, samples were mixed with ice-cold saline in a ratio of 1 : 9. Subsequently, the mixture was crushed and centrifuged at 8000 rpm for 10 min at 4°C (Eppendorf centrifuge 5810R, Germany). After centrifugation, the supernatant in the tube was taken and aliquoted into 200 *μ*l centrifuge tubes and finally stored at -80°C for analysis. Parameters of antioxidant capacity included the activities of total antioxidant capacity (T-AOC), total superoxide dismutase (T-SOD), glutathione (GSH), and the concentration of malondialdehyde (MDA) as well as enzymatic activities of hydroxyl radical scavenging activity. The parameters above were totally analyzed by assay kits (Nanjing Jiancheng Bioengineering Institute, China).

### 2.7. Analysis of Amino Acid Composition

About 0.1 g muscle samples were weighed into a glass tube then add 5 ml of 6 N HCL, blow out excess air with nitrogen, seal the bottle, and put it in a sand bath of 110°C for 24 h. Pour out the liquid in the bottle and filter the impurities and then maintain a constant volume to a 50 ml volumetric flask, take 1 ml and dry it with nitrogen, and add 0.05 N HCL to dissolve it. And the residue was filtered with a 0.2 *μ*m nylon membrane. After the preprocessing, the values were analyzed through L-8900 amino acid analyzer (Hitachi, Japan), and the detailed procedure was referred to the previous study [30].

### 2.8. Analysis of Fatty Acid Profile

The specific method for the determination of fatty acid composition in the hepatopancreas and diets was referred to previous studies [28, 31]. 1 ml methyl tricosanoate (1 mg ml^−1^) was added to a glass tube with a screwcap and blown dry with nitrogen. Next, about 100 mg of the sample was weighed per tube and 3 ml BHT (0.25 mg ml^−1^, 0.025 g BHT, 1 ml H_2_SO_4_, and 99 ml CH_3_OH) solution was added to the glass tube. The glass tubes were then placed in an 80°C water bath for 3 hours and shaken every 20 minutes. After cooling the glass tube, add 1 ml n-hexane and 1 ml ultrapure water and shake for 1 minute. After stratification, the upper transparent liquid was extracted and concentrated with nitrogen and finally stored in 0.5 ml of n-hexane at -20°C until analysis. Results of fatty acid were measured by a gas chromatograph mass spectrometer (Agilent Technologies, CA, USA).

### 2.9. RT-PCR Analysis

Hepatopancreas samples stored at -80°C were thawed and transferred to 1 ml TRIzol reagent (Invitrogen, USA) for RNA extraction, followed by agarose gel electrophoresis and NanoDrop 2000 spectrophotometer (NanoDrop Technologies, USA) to assess RNA quality and quantity. According to the instructions of HiScript RT SuperMix Reagent Kit (Vazyme, China), synthesize cDNA for RT-qPCR. The specific primers were all designed from the base of transcriptome (PRJNA432636) [32] and were used for analysis of RT-qPCR (Table 4). The total reaction system for RT-qPCR includes 0.8 *μ*l of primer, 0.8 *μ*l cDNA template, 8.4 *μ*l DEPC-water, and 10 *μ*l SYBR Green premix. The RT-qPCR conditions and calculation method of mRNA expression levels were referred to the previous study [30].

### 2.10. Calculations

Percent weight gain (PWG, %) = 100 × (*W*_*t*_ − *W*_*i*_)/*W*_*i*_.

Specific growth rate (SGR, %day^−1^) = 100 × (ln*W*_*t*_ − ln*W*_*i*_)/*t*.

Feed efficiency (FE) = (*W*_*t*_ + *W*_*d*_ − *W*_*i*_) (g, wet weight)/feed intake (g, dry weight).

Survival (%) = 100 × (final number of crab)/(initial number of crab).

Comments: *W*_*t*_: final body weight (g); *W*_*i*_: initial body weight (g); *W*_*d*_: dead body weight (g); *t*: experimental duration.

The results with three replicates were manifested as the means ± S.E.M. All statistics were evaluated by one-way ANOVA followed by Tukey's multiple-range test to determine significant differences between the treatment groups; *P* < 0.05 was set for the level of significance. Analyses of data were conducted using SPSS 20.0 (SPSS, USA).

## 3. Results

### 3.1. Growth Performance, Feed Utilization, and Survival

As is shown in Table 5, survival and feed efficiency (FE) showed no significance with the influence of dietary FM replacement with KM (*P* > 0.05). Crabs fed with the KM30 diet had lower final weight (FW), percent weight gain (PWG), and specific growth rate (SGR) than those fed with the KM0 and KM10 diets (*P* < 0.05).

### 3.2. Proximate Composition in the Muscle and the Hepatopancreas

As is shown in Table 6, the highest content of protein in the hepatopancreas was manifested in crabs fed with the KM30 diet (*P* < 0.05), while contents of moisture and lipid were not significantly influenced by the experimental diets (*P* > 0.05). Crabs fed with the KM20 and KM30 diets had higher lipid content in the muscle than those fed with the control diet (*P* < 0.05).

### 3.3. Hematological Characteristics

As is exhibited in Table 7, total protein (TP), albumin (ALB), low density lipoprotein (LDL), and triglyceride (TG) in the hemolymph were notably influenced by substitution levels of FM with KM (*P* < 0.05). The lowest concentration of TP, ALB, and TG was shown in the crabs fed with the KM30 diet (*P* < 0.05) and crabs fed with the KM20 diet had the highest concentration of LDL within all the treatment groups (*P* < 0.05). Nevertheless, no distinctions were presented in the concentrations of high density lipoprotein (HDL), total cholesterol (T-CHO), and glucose (GLU) (*P* > 0.05).

### 3.4. Antioxidant Parameters in the Hemolymph and the Hepatopancreas

The effect of dietary replacement of FM with KM on antioxidant parameters in the hemolymph and the hepatopancreas is presented in Table 8. In the hemolymph, crabs fed with the KM10 and KM20 diets showed the highest activities of T-AOC and hydroxyl radical scavenging within all dietary treatments (*P* < 0.05). Crabs fed with the KM30 diet had significantly higher activity of total superoxide dismutase (T-SOD) than those fed with other diets (*P* < 0.05), and crabs fed with the KM10 and KM20 diets had the highest glutathione (GSH) concentration than crabs fed with other diets (*P* < 0.05). Meanwhile, crabs fed with the diet without KM supplementation had significantly higher malondialdehyde (MDA) than crabs fed with diets with KM supplementation (*P* < 0.05).

In the hepatopancreas, crabs fed with the KM30 diet had the highest concentration of GSH and activities of T-AOC and hydroxyl radical scavenging among all treatments (*P* < 0.05). Crabs fed with the KM10 and KM20 diets had significantly higher total superoxide dismutase (T-SOD) than crabs fed with other diets (*P* < 0.05). Furthermore, with dietary substitution of FM with KM increasing from 0% to 30%, the concentration of MDA notably decreased (*P* < 0.05).

### 3.5. Amino Acid Profile

As is shown in Table 9, crabs fed with the KM30 diet had the highest glycine content in the muscle within all the treatment groups (*P* < 0.05), whereas there were no distinctions in other nonessential amino acids and all essential amino acids among all the treatment groups.

### 3.6. Fatty Acid Composition

As is presented in Table 10, the contents of 14 : 0 and 16 : 0 in the hepatopancreas were notably increased with the increasing replacement of FM with KM, and the highest content of saturated fatty acids occurred in the KM30 group (*P* < 0.05). Crabs fed with the KM30 diet had significantly lower contents of 18:2n-6, 20:4n-6, and total n-6 PUFA than those fed with other diets (*P* < 0.05). Crabs fed with the KM30 diet had significantly higher contents of EPA and 22:5n-3 than those fed with other diets (*P* < 0.05), while crabs fed with the KM30 diet had the lowest contents of 18:3n-3 and DHA in the hepatopancreas. With the increase of dietary replacement of FM with KM, the ratios of DHA to EPA in the hepatopancreas significantly decreased, whereas no significant differences were observed in the composition of MUFA and n-3 PUFA among all the treatment groups (*P* > 0.05).

### 3.7. Hepatopancreas Coloration

**Figure 1:**
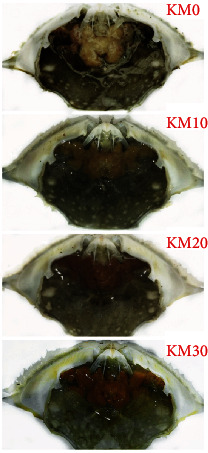
Hepatopancreas color comparison of swimming crab fed with diets with different replacement of fish meal with krill meal.

The effects of different replacements of FM with KM on the hepatopancreas coloration are presented in Figure 1. With the substitution level of FM with KM gradually increasing from 0% to 30%, the color of the hepatopancreas changed from pale white to red.

### 3.8. mRNA Levels Involved into TOR Pathway in the Hepatopancreas

**Figure 2:**
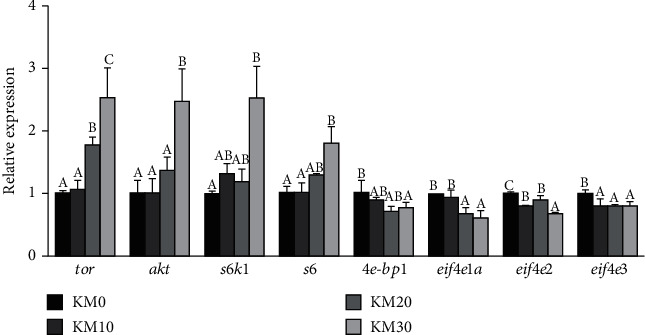
Effects of different replacement levels of fish meal with krill meal on expression levels of genes involved into mTOR signaling pathway of *Portunus trituberculatus* hepatopancreas. The gene expression of KM0 was set at 1. Values are means (*n* = 3), and S.E.M. is expressed as a vertical T bar. The mean value of different letters in the same gene was different (*P* < 0.05) (Tukey's test).

As is shown in Figure 2. mRNA levels of *tor*, *akt*, *s6k1*, and *s6* were notably activated in the crabs fed with the KM30 diets (*P* < 0.05) while expression of *4e-bp1*, *eif4e1a*, *eif4e2*, and *eif4e3* was the lowest among all the treatment groups (*P* < 0.05).

### 3.9. mRNA Expression Levels Involved into Antioxidation in the Hepatopancreas

**Figure 3:**
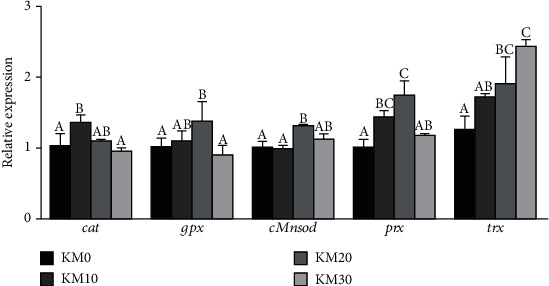
Effects of different replacement levels of fish meal with krill meal on relative mRNA expression levels of antioxidant genes in the hepatopancreas of *Portunus trituberculatus*. The gene expression of KM0 was set at 1. Values are means (*n* = 3), and S.E.M. is expressed as a vertical T bar. The mean value of different letters in the same gene was different (*P* < 0.05) (Tukey's test).

As is presented in Figure 3, crabs fed with the KM20 diet had higher expression of *cMnsod*, *gpx*, and *prx* in the hepatopancreas than those fed with other diets, and the lowest expression of *gpx*, *cMnsod*, and *prx* was presented in crabs fed with the KM0 diet (*P* < 0.05). Meanwhile, gene of *trx* was notably activated with dietary replacement of FM with KM increasing from 0% to 30% (*P* < 0.05). Moreover, crabs fed with the KM10 diet had significantly higher expression of *cat* than those fed with the KM0 and KM30 diets (*P* < 0.05).

## 4. Discussion

Previous studies have illustrated that dietary krill meal supplementation could enhance the growth performance of fish [33–35] and crustaceans [3, 19] and promote feed utilization in crustacean diets as an attractant or a growth promoter [33, 36, 37]. As was observed in the present study, crabs fed with the KM10 diet had higher PWG and SGR than those fed with the KM30 diet. The results of previous studies reported that dietary 10% KM supplementation can be used as the most suitable diet for growth performance in the Pacific white shrimp (*Litopenaeus vannamei*) [3]. Similar results were reported in the tiger shrimp (*Penaeus monodon*) [34]. Gao et al. [19] indicated that the optimum replacement proportion of FM with KM was 50% for red swamp crayfish (*Procambarus clarkia*) (the amount of FM in the control diet was 43%). In contrast, Nunes et al. [38] demonstrated that dietary KM supplementation made no positive influence on the growth performance of *L. vannamei*, probably because of the dietary low KM supplementation. In the present study, more than 20% replacement of FM with KM resulted in a significant decrease in PWG and SGR. The results demonstrated that excessive KM could inhibit the growth performance of swimming crabs. Different studies have come to different or even opposite conclusions on the evaluation of the effect of dietary KM supplementation on growth for crustaceans. The reason may be due to the different processing technology and nutrient composition of KM, dietary formulation especially plant protein sources, species, different animal sizes, and culture conditions. Moreover, the fluorine content in KM is also a major factor for dosage of KM. In the present study, with the increasing replacement level of FM with KM, fluorine content in experimental diets increased from 27.16 to 265.30 mg/kg. How the fluorine content in KM affects the growth of crustaceans is still unclear and the related mechanism needs further study.

In this study, results indicated that crabs fed with the diet without KM supplementation had the lowest protein content in the hepatopancreas and lipid content in the muscle among all the treatment groups. Compared with regular FM, Gao et al. [19] indicated that KM could induce the improvement of amino acid patterns by the increasing of protein biosynthesis in red swamp crayfish. Wang et al. [25] proved that the dietary replacement levels of FM with soy protein concentrate could significantly affect the proximate composition in the hepatopancreas of swimming crabs. A previous study exhibited that the content of crude lipid of whole crab was reduced with the substitution rate of FM with low-gossypol cottonseed protein concentrate exceeding 40% for juvenile swimming crab [29]. Different studies could draw diametrically different conclusions on the same study species (swimming crab) which may be due to different protein sources, such as soy protein concentrate and low-gossypol cottonseed protein concentrate [25, 26]. The juvenile swimming crab cannot synthesize essential amino acids itself, so they must obtain them from the diets [27]. In this study, results indicated that the content of essential amino acids (EAA) in the muscle was observed without a significant influence. Dietary substitution of FM with plant protein sources showed similar results of no distinctions in EAA contents in the muscle for rainbow trout (*Oncorhynchus mykiss*) [39], red claw crayfish (*Cherax quadricarinatus*) [40], and Atlantic cod (*Gadus morhua*) [41]

Liver or hepatopancreas had the function of lipid storage. Generally speaking, fatty acid profiles in the liver or the hepatopancreas reflect the dietary fatty acid profiles [28]. In the present study, with the increasing replacement level of FM with KM, eicosapentaenoic acid (EPA) in the hepatopancreas significantly increased. On the contrary, docosahexaenoic acid (DHA) in the hepatopancreas decreased with the increasing level of the replacement of FM with KM. Correspondingly, similar results of the composition of DHA and EPA in the hepatopancreas were reported in previous studies on dietary lipid sources of marine crustaceans [42–45]. Mammal-related studies demonstrated that a lower ratio of DHA to EPA seems to be more beneficial to alleviate liver damage in mice caused by a high-fat diet, and when the ratio of DHA to EPA is 1 : 2, it could reduce factors for inflammation [46].

The antioxidant system in animals is composed of enzymatic molecules such as catalase (CAT), glutathione peroxidase (GSH-PX), superoxide dismutase (SOD), and nonenzymatic molecules such as glutathione (GSH) and AX [15]. To ensure the intracellular redox balance, the tripeptide glutathione (GSH), consisting of glutamate, cysteine, and glycine, played a key role as the major factor [7]. Counterbalance such as enzymes (SOD and T-AOC) and functionalized molecules (GSH) could play an important role in eliminating oxidative stress for crabs [28, 47]. In the present study, the activities of T-AOC, SOD, and GSH significantly increased in the hemolymph and the hepatopancreas with the increasing level of replacement of FM with KM. It is speculated that AX in KM played a key role in activating antioxidant system [15]. Since malondialdehyde (MDA) is a product of lipid peroxidation, its concentration in tissues is usually used to estimate the degree of lipid peroxidation [28]. In this study, content of MDA in the hemolymph and the hepatopancreas was presented prominently lower in crabs fed with the dietary supplementation with KM. Results suggested that dietary KM supplementation could reduce lipid peroxidation. Hydroxyl free radicals are one of the reactive oxygen species produced by the body, which can easily react with biomolecules such as amino acids and proteins and result in the disorders of physiological metabolism [48]. Therefore, removal of hydroxyl radicals can reduce the exposure of organisms to harsh conditions or diseases [49, 50]. In the present study, results demonstrated that the increase of KM replacement level significantly improved the hydroxyl radical scavenging activity of swimming crabs.

AX is a ketocarotenoid; in addition to its physiological effects on enhancing immunity, anticancer, and scavenging free radicals in the body, AX is also the main pigment in aquatic animals [51]. It has been reported that the addition of AX exhibited a positive effect on the redness of the cooked carapace of *P. trituberculatus* [52], and similar results were also performed in *L. vannamei* [53]. The content of AX in the hepatopancreas was not analyzed in this experiment, but it was clearly observed that the content of dietary KM, especially the AX in KM played a great role in the hepatopancreas color changing from pale to red. Yao et al. [3] demonstrated that the AX content in the hepatopancreas was positively correlated with the dietary supplement of KM because it is rich in AX, and dietary AX can accumulate in the tissue of shrimp [54].

mTOR belongs to a typical serine/threonine protein kinase of PI3K-related kinases. mTOR complexes 1 (mTORC1) and 2 (mTOR2) are two complexes formed by the interaction of mTOR with multiple proteins. The mTORC1 reacts with insulin and insulin-like peptides (ILPs) via the PI3K pathway, and AKT could be phosphorylated by PI3K through activating phosphoinositide-dependent kinase-1; then, mTORC1 could be activated by AKT through phosphorylating tuberous sclerosis complex 2/ PRAS40 [55]. Previous studies have indicated that continuous ecdysis steroid synthesis and secretion in ecdysis glands of insects and crustaceans depend on mTOR protein synthesis [56, 57]. The main rate-limiting step of protein synthesis is regulated by the mTOR signaling pathway; mTORC1 directly phosphorylates downstream target proteins ribosomal protein S6 kinase1 (S6K1) and eukaryotic initiation factor 4E binding protein-1 (4E-BP1), and activated S6K1 can phosphorylate ribosomal protein S6 (S6), thereby promoting a variety of proteins involved in the protein synthesis and the translation initiation extension; transcription is activated, and the phosphorylated 4E-BP1 is separated from the eukaryotic translation initiation factor 4E (eIF4E) to release the inhibition of translation [58–62]. However, previous studies on insulin/mTOR pathway and its function in crustacean physiology and metabolism are incomplete. In the present study, expression of *akt* was upregulated; consequently, it could activate the TOR pathway which led to the upregulation of expression of *tor*, and the highest expression of *tor* presented in crabs fed with diet with 30% substitution of FM with KM. It is speculated that dietary KM supplementation could enhance the secretion of insulin-like peptides (ILPs) and thus upregulate the expression of genes involved in the TOR pathway. Moreover, downstream genes (*s6k1* and *s6*) showed the same trend that is also consistent with previous studies [55].

In the present study, results indicated that increasing substitution of FM with KM could upregulate the expression of genes involved in antioxidant such as *cat*, *gpx*, and *cMnsod*; expression of genes of *cat* and *gpx* were downregulated when replacement of FM with KM exceeded 20%. In this study, the mRNA levels of *prx* and *trx* were stimulated in crabs fed with diets with 20% and 30% replacement of FM with KM; crabs fed with the diet without KM supplementation had the lowest expression of *prx* and *trx* among all the treatment groups. It may suggest that dietary KM supplementation could stimulate the levels of antioxidant parameters including genes and enzymes in the hepatopancreas of swimming crab and catalyze ROS to less reactive species [63].

In summary, this study demonstrated that dietary 10% replacement of FM with KM could improve growth performance, enhance the antioxidant capacity, and stimulate the mRNA expression levels related to protein synthesis and antioxidant of swimming crab. The results indicated that KM could be used as a good protein source for swimming crab; KM has a relative balance of amino acids and fatty acids similar to FM; AX in KM could promote antioxidant capacity in the hepatopancreas and improve the color of hepatopancreas. Nevertheless, the fluorine content in KM is an important factor affecting its addition in aquafeed. The interaction between protein source and growth regulation mechanism needs to be further studied; the results could provide a basis for the selection and utilization of feed ingredients, especially protein sources, during the growth stage of swimming crab.

## Figures and Tables

**Table 1:** Ingredients and proximate composition of the experimental diets (dry matter basis, %).

Ingredients	Replacement levels of FM with KM			
	0%	10%	20%	30%
Peru fish meal	40.00	36.00	32.00	28.00
Soybean meal	20.00	20.00	20.00	20.00
Krill meal	0.00	4.78	9.56	14.34
Yeast brewers	3.00	3.00	3.00	3.00
Wheat flour	22.89	22.89	22.89	22.89
Lysine	0.04	0.03	0.02	0.02
Methionine	0.09	0.07	0.06	0.04
Fish oil	2.85	2.39	1.94	1.48
Soybean lecithin	2.00	2.00	2.00	2.00
Vitamin premix^a^	1.00	1.00	1.00	1.00
Mineral premix^b^	2.00	2.00	2.00	2.00
Ca(H_2_PO_4_)_2_	2.00	2.00	2.00	2.00
Choline chloride	0.30	0.30	0.30	0.30
Sodium alginate	2.00	2.00	2.00	2.00
Cellulose	1.83	1.54	1.23	0.93
Proximate composition (%)				
Dry matter	89.52	88.84	91.58	91.34
Ash	11.09	10.78	10.89	10.95
Lipid	8.99	8.90	9.03	8.89
Protein	44.94	45.99	45.23	45.59
Fluorine content (mg/kg)	27.16	94.06	153.81	265.30

^a, b^The preparation of mineral mixture and vitamin mixture was on the basis of Wang et al. [25].

**Table 2:** The amino acid profile of the experimental diets (g/kg, dry matter).

Amino acids	Replacement levels of FM with KM			
	0	10%	20%	30%
EAA^a^				
Thr	18.26	18.69	18.65	18.40
Val	21.66	21.90	21.54	21.00
Met	11.49	11.72	11.70	11.21
Ile	17.48	18.54	18.48	18.53
Leu	33.13	34.93	34.93	34.16
Phe	18.55	19.45	19.90	19.86
Lys	29.16	30.01	30.19	29.07
His	11.02	10.47	10.46	9.96
Arg	25.59	26.57	26.49	26.14
NEAA^b^				
Asp	37.51	38.07	38.15	37.74
Ser	18.17	18.46	18.50	18.08
Glu	67.37	67.16	67.13	66.14
Gly	17.69	18.27	18.19	19.84
Ala	19.57	21.72	21.44	20.71
Cys	7.24	6.59	5.98	6.16
Tyr	13.12	13.57	13.50	13.77
Pro	55.33	54.78	54.46	55.41
∑EAA	186.34	192.28	192.34	188.33
∑NEAA	236.00	238.62	237.36	237.83
TAA^c^	422.34	430.89	429.70	426.16
∑EAA/TAA	0.44	0.45	0.45	0.44

^a^EAA: essential amino acids; ^b^NEAA: nonessential amino acids; ^c^TAA: total amino acids.

**Table 3:** Fatty acid composition of experimental diets (mg/g, based on dry matter).

Fatty acids	Replacement levels of FM with KM			
	0	10%	20%	30%
14 : 0	2.15	2.99	3.64	4.46
16 : 0	13.28	12.83	13.38	14.56
18 : 0	4.20	3.85	3.76	3.66
20 : 0	0.20	0.16	0.15	0.12
∑SFA^a^	19.83	19.84	20.92	22.80
16:1n-7	3.15	4.16	4.54	4.99
18:1n-9	7.40	6.84	7.12	7.57
20:1n-9	0.85	0.42	0.47	0.53
22:1n-11	0.11	0.14	0.20	0.26
∑MUFA^b^	11.51	11.55	12.33	13.35
18:2n-6	15.73	13.07	13.03	13.56
18:3n-6	0.06	0.07	0.07	0.09
20:2n-6	0.12	0.08	0.08	0.07
20:4n-6	0.59	0.57	0.55	0.53
∑n-6PUFA^c^	16.51	13.80	13.73	14.25
18:3n-3	2.24	1.73	1.77	1.91
18:4n-3	0.71	0.69	0.85	1.06
20:4n-3	0.26	0.27	0.26	0.25
20:5n-3	4.48	7.29	8.33	9.32
22:5n-3	0.58	1.05	1.00	0.94
22:6n-3	9.88	7.93	7.93	7.84
∑n-3PUFA^d^	18.14	18.96	20.14	21.32
Total	65.98	64.16	67.12	71.72
EPA+DHA	14.37	15.22	16.26	17.16
DHA/EPA^e^	2.21	1.09	0.95	0.84

^a^SFA: saturated fatty acids; ^b^MUFA, mono-unsaturated fatty acids; ^c^n-6 PUFA: n-6 polyunsaturated fatty acids; ^d^n-3 PUFA: n-3 polyunsaturated fatty acids; ^e^DHA/EPA: the ratio of 22:6n-3 to 20:5n-3.

**Table 4:** Real-time quantitative PCR primer-related genes of swimming crab (*Portunus trituberculatus*).

Genes names	Primers		Size (bp)	Access no.
	Forward (5′-3′)	Reverse (5′-3′)		
*β-actin*	GAAGTAGCCGCCCTGGTTGT	GAATACCTCGCTTGCTCTGC	184	[25]
*cat* ^a^	GCTCTGACCCTGACTATGCT	CGACCAACAGGAATTAGCGG	180	FJ152102.2
*gpx* ^b^	TACCGTTCAGCAAGTACCGT	GTGGTGTTTTCCTGGTGACC	171	KY216076.1
*cMnsod* ^c^	GATCCATCACACCAAGCACC	GTTCTCCTCCAGCTTCAGGT	193	FJ031018.1
*prx* ^d^	TGACCAGCAACATGAGCAAC	CAATCTTGCGGAACTCCTCG	215	FJ174664.1
*trx* ^e^	GAAAGGGCGAGAAGGTCCA	CTACTCCCCAATGAGCCGAT	188	OP081649
*tor^f^*	TGTGGACATAGGGCAAACTG	GACCGCTTCACCAAATCATC	174	[25]
*akt^g^*	GGACTACGAGGCACCAAGAA	TGGACCACTTCATCACGCTC	179	[25]
*s6k1^h^*	CGCCCCTCAGATTTCCAGT	TCTCAGCCTTTGTGTGCG	175	[25]
*s6^i^*	CACTAACAACCGTGTGCGAC	ACCCTTCTTGATGATGACCAG	148	[25]
*4e-bp1^j^*	GGCTGAACTTCCAAATGACT	TTCTTGGGTGGGGTCTTG	142	[25]
*eif4e1a^k^*	TGAACAAGCAGCAGCGAG	TGACCACAGCACCACACA	114	[25]
*eif4e2*	GCTTTCAGGAGGACATCATC	TGAGGGAGTCATTGTGAGTCT	144	[25]
*eif4e3*	CCCTGGACCTTCTGGATTGA	TCCTGTTACCCCTCATCAAG	181	[25]

^a^ *cat*: catalase; ^b^*gpx*: glutathione peroxidase; ^c^*cMnsod*: cytosolic manganese superoxide dismutase; ^d^*prx*, thioredoxin peroxidase; ^e^*trx*: thioredoxin; ^f^*tor*: target of rapamycin; ^g^*akt*: protein kinases B; ^h^*s6k1*: ribosomal protein S6 kinase1; ^i^*s6*: ribosomal protein S6; ^j^*4e-bp1*: eukaryotic initiation factor 4E binding protein-1; ^k^*eif4e*: eukaryotic translation initiation factor 4E.

**Table 5:** Growth performance, feed utilization, and survival of *Portunus trituberculatus* fed with diets with different replacement levels of FM with KM.

Items	Replacement levels of FM with KM			
	0	10%	20%	30%
IBW^a^ (g)	5.68 ± 0.05	5.59 ± 0.02	5.62 ± 0.04	5.62 ± 0.03
Final weight (g)	59.86 ± 2.00^b^	63.23 ± 1.26^b^	55.44 ± 2.88^ab^	52.10 ± 2.18^a^
PWG (%)^b^	953.48 ± 27.61^b^	1030.52 ± 25.11^b^	886.39 ± 43.73^ab^	826.98 ± 43.49^a^
SGR (%/d) ^c^	4.20 ± 0.05^b^	4.33 ± 0.04^b^	4.08 ± 0.08^ab^	3.97 ± 0.08^a^
Survival (%)	86.67 ± 5.77	90.00 ± 10.00	86.67 ± 5.77	83.33 ± 5.77
FE (%/d) ^d^	0.44 ± 0.03	0.45 ± 0.02	0.43 ± 0.03	0.43 ± 0.03

The mean values of three replicates ± S.E.M. Difference of superscript letters in the same row indicates statistical significance (*P* < 0.05). ^a^IBW: initial body weight; ^b^PWG: percent weight gain; ^c^SGR: specific growth rate; ^d^FE: feed efficiency.

**Table 6:** Proximate composition in the hepatopancreas and muscle of *Portunus trituberculatus* fed with the experimental diets for 8 weeks (%, based on wet weight).

Items	Replacement levels of FM with KM			
	0	10%	20%	30%
Hepatopancreas				
Moisture	66.31 ± 0.01	66.25 ± 1.69	66.25 ± 0.27	66.45 ± 0.99
Protein	11.30 ± 0.06^a^	11.58 ± 0.52^a^	12.21 ± 0.16^ab^	13.19 ± 0.41^b^
Lipid	15.46 ± 0.43	16.38 ± 1.62	14.94 ± 0.68	14.80 ± 0.85
Muscle				
Moisture	78.54 ± 0.07	78.38 ± 0.13	78.53 ± 0.18	78.67 ± 0.01
Protein	17.85 ± 0.06	17.89 ± 0.12	17.97 ± 0.13	18.19 ± 0.09
Lipid	0.96 ± 0.04^a^	1.11 ± 0.03^ab^	1.23 ± 0.05^b^	1.19 ± 0.03^b^

The mean values of three replicates ± S.E.M. Difference of superscript letters in the same row indicates statistical significance (*P* < 0.05).

**Table 7:** Hemolymph biochemical indices of *Portunus trituberculatus* fed with different experimental diets for 8 weeks.

Items	Replacement levels of FM with KM			
	0	10%	20%	30%
ALB^a^ (g/L)	5.95 ± 0.38^b^	5.90 ± 0.06^b^	5.45 ± 0.03^b^	4.25 ± 0.09^a^
TP^b^ (g/L)	63.61 ± 5.27^b^	58.01 ± 0.24^b^	55.82 ± 0.50^b^	50.19 ± 0.59^a^
LDL^c^ (mmol/L)	0.13 ± 0.01^a^	0.15 ± 0.01^ab^	0.20 ± 0.01^b^	0.14 ± 0.02^ab^
HDL^d^ (mmol/L)	0.15 ± 0.01	0.16 ± 0.01	0.18 ± 0.01	0.14 ± 0.01
TG^e^ (mmol/L)	0.09 ± 0.03^ab^	0.10 ± 0.03^b^	0.09 ± 0.06^ab^	0.05 ± 0.03^a^
T-CHO^f^ (mmol/L)	0.25 ± 0.03	0.25 ± 0.03	0.30 ± 0.06	0.25 ± 0.03
GLU^g^ (mmol/L)	2.87 ± 0.34	2.68 ± 0.06	3.04 ± 0.08	2.09 ± 0.06

The mean values of three replicates ± S.E.M. Difference of superscript letters in the same row indicates statistical significance (*P* < 0.05). ^a^ ALB, albumin; ^b^ TP, total protein; ^c^ LDL, low denstiy lipoprotein; ^d^ HDL, high density lipoprotein; ^e^ TG, triglyceride; ^f^ T-CHO, total cholesterol; ^g^ GLU, glucose.

**Table 8:** Antioxidant parameters in the hemolymph and the hepatopancreas of *Portunus trituberculatus* fed with different experimental diets for 8 weeks.

Items	Replacement levels of FM with KM			
	0	10%	20%	30%
Hemolymph				
T-AOC^a^ (U/mL)	0.97 ± 0.03^a^	1.40 ± 0.08^b^	1.48 ± 0.12^b^	1.00 ± 0.06^a^
SOD^b^ (U/mL)	54.88 ± 4.67^a^	61.02 ± 1.23^a^	68.72 ± 7.28^ab^	83.90 ± 0.96^b^
MDA^c^ (nmol/mL)	4.46 ± 0.08^b^	3.08 ± 0.07^a^	2.39 ± 0.24^a^	2.78 ± 0.01^a^
GSH^d^ (mgGSH/L)	10.56 ± 0.67^a^	14.46 ± 0.62^b^	13.89 ± 0.27^b^	13.01 ± 0.96^ab^
Hydroxyl radical scavenging activity (U/L)	196.94 ± 12.53^a^	246.92 ± 0.67^b^	256.80 ± 9.29^b^	194.19 ± 5.73^a^
Hepatopancreas				
T-AOC (mgpro/mL)	3.11 ± 0.03^a^	3.61 ± 0.20^ab^	4.09 ± 0.13^b^	4.10 ± 0.10^b^
SOD (U/mgprot)	5.60 ± 0.17^a^	5.55 ± 0.05^a^	8.29 ± 0.15^b^	7.55 ± 0.24^b^
MDA (nmol/mgprot)	2.86 ± 0.08^c^	2.39 ± 0.12^b^	2.07 ± 0.02^ab^	1.87 ± 0.04^a^
GSH (mgGSH/gprot)	3.74 ± 0.16^a^	3.74 ± 0.36^a^	3.99 ± 0.14^a^	6.11 ± 0.14^b^
Hydroxyl radical scavenging activity (U/mgprot)	321.72 ± 13.09^a^	377.70 ± 7.95^b^	376.06 ± 8.77^b^	428.13 ± 4.48^c^

The mean values of three replicates ± S.E.M. Difference of superscript letters in the same row indicates statistical significance (*P* < 0.05). ^a^T-AOC: total antioxidant capacity; ^b^SOD: total superoxide disumutase; ^c^MDA: malondialdehyde; ^d^GSH: glutathione.

**Table 9:** Amino acid composition in the muscle of *Portunus trituberculatus* fed with diets with different replacement levels of FM with KM.

Amino acids (g kg^−1^)	Replacement levels of FM with KM			
	0	10%	20%	30%
EAA				
Thr	29.46 ± 0.39	29.19 ± 0.31	29.08 ± 0.33	28.94 ± 0.12
Val	31.62 ± 0.50	29.72 ± 0.20	31.01 ± 0.23	29.66 ± 0.90
Met	16.72 ± 0.60	15.38 ± 0.64	17.28 ± 0.52	16.94 ± 1.27
Ile	29.13 ± 0.39	28.07 ± 0.37	28.03 ± 0.40	28.54 ± 0.18
Leu	55.12 ± 0.63	54.06 ± 0.64	53.92 ± 0.7	54.38 ± 0.18
Phe	26.83 ± 0.53	26.73 ± 0.54	26.31 ± 0.35	26.57 ± 0.23
Lys	58.25 ± 0.53	57.30 ± 0.54	56.86 ± 0.35	57.42 ± 0.23
His	14.47 ± 0.46	14.80 ± 0.44	14.29 ± 0.14	14.39 ± 0.22
Arg	85.82 ± 1.59	86.17 ± 0.98	85.52 ± 1.65	86.42 ± 0.80
NEAA				
Asp	61.02 ± 0.46	59.54 ± 0.86	58.65 ± 0.40	58.84 ± 0.19
Ser	27.25 ± 0.29	26.89 ± 0.29	26.95 ± 0.31	27.03 ± 0.18
Glu	103.27 ± 1.09	101.65 ± 1.17	99.20 ± 1.21	102.04 ± 1.50
Gly	48.46 ± 0.94^a^	46.98 ± 2.25^a^	50.27 ± 2.55^ab^	57.88 ± 1.41^b^
Ala	39.43 ± 1.18	36.76 ± 1.33	38.21 ± 0.30	40.15 ± 0.93
Cys	9.91 ± 0.27	8.91 ± 0.38	10.20 ± 0.06	9.51 ± 0.58
Tyr	24.77 ± 0.63	24.47 ± 0.43	24.16 ± 0.32	24.53 ± 0.09
Pro	81.70 ± 0.58	81.02 ± 1.53	79.89 ± 1.80	80.75 ± 1.29
∑EAA	292.29 ± 0.74	287.36 ± 2.17	288.38 ± 4.35	288.89 ± 1.93
∑NEAA	395.81 ± 2.82	386.22 ± 0.42	387.54 ± 5.51	400.73 ± 2.87
TAA	688.10 ± 2.79	673.58 ± 1.76	675.92 ± 8.78	689.62 ± 4.57
∑EAA/TAA	0.42 ± 0.00	0.43 ± 0.00	0.43 ± 0.00	0.42 ± 0.00

The mean values of three replicates ± S.E.M. Difference of superscript letters in the same row indicate statistical significance (*P* < 0.05).

**Table 10:** Fatty acid composition in the hepatopancreas of *Portunus trituberculatus* fed with diets with different replacement levels of FM with KM.

Fatty acids (mg g^−1^)	Replacement levels of FM with KM			
	0	10%	20%	30%
14 : 0	5.13 ± 0.42^a^	5.54 ± 0.10^ab^	7.26 ± 0.29^bc^	7.57 ± 0.72^c^
16 : 0	32.29 ± 0.27^a^	31.41 ± 0.20^a^	32.5 ± 0.34^a^	34.45 ± 0.14^b^
18 : 0	18.28 ± 0.32	19.91 ± 1.18	17.2 ± 0.32	17.86 ± 0.19
20 : 0	1.29 ± 0.07	1.00 ± 0.12	1.06 ± 0.10	0.92 ± 0.03
∑SFA^a^	56.99 ± 0.82^a^	57.86 ± 1.33^ab^	58.02 ± 0.94^bc^	60.79 ± 0.69^c^
16:1n-7	8.63 ± 0.18^a^	8.56 ± 0.10a	10.18 ± 0.05^b^	10.6 ± 0.09^b^
18:1n-9	26.87 ± 1.26	27.59 ± 1.50	26.94 ± 1.63	24.96 ± 1.33
20:1n-9	6.06 ± 0.07^c^	5.01 ± 0.20^b^	4.25 ± 0.16^a^	4.38 ± 0.03^a^
22:1n-11	0.73 ± 0.03^a^	0.72 ± 0.09^a^	1.22 ± 0.06^b^	1.28 ± 0.10^b^
∑MUFA^b^	42.30 ± 1.50	41.88 ± 1.69	42.60 ± 1.61	41.22 ± 1.31
18:2n-6	34.33 ± 0.08^b^	38.82 ± 0.42^c^	31.48 ± 0.92^ab^	28.86 ± 1.49^a^
18:3n-6	0.18 ± 0.02	0.18 ± 0.03	0.17 ± 0.01	0.17 ± 0.02
20:2n-6	5.76 ± 0.25	5.55 ± 0.31	4.91 ± 0.16	4.81 ± 0.25
20:4n-6	2.51 ± 0.13^b^	2.26 ± 0.13^ab^	2.33 ± 0.03^b^	1.85 ± 0.06^a^
∑n-6 PUFA^c^	42.78 ± 0.36^b^	46.81 ± 0.31^c^	38.89 ± 0.80^ab^	35.69 ± 1.45^a^
18:3n-3	5.44 ± 0.35^c^	4.35 ± 0.19^b^	3.80 ± 0.18^ab^	3.15 ± 0.17^a^
18:4n-3	1.07 ± 0.15	0.95 ± 0.27	1.08 ± 0.08	1.25 ± 0.17
20:4n-3	0.72 ± 0.08	0.71 ± 0.11	0.71 ± 0.03	0.65 ± 0.04
20:5n-3	11.35 ± 0.53^a^	11.56 ± 0.44^a^	16.34 ± 0.10^b^	17.01 ± 0.01^b^
22:5n-3	1.60 ± 0.06^a^	2.22 ± 0.20^b^	2.78 ± 0.06^c^	2.43 ± 0.04^bc^
22:6n-3	27.25 ± 1.41^b^	22.33 ± 1.75^ab^	19.89 ± 0.74^a^	17.28 ± 0.67^a^
∑n-3 PUFA^d^	46.85 ± 2.40	41.62 ± 2.07	44.38 ± 1.04	42.00 ± 0.96
Total	193.63 ± 4.52	186.19 ± 5.12	187.71 ± 3.30	175.96 ± 4.28
EPA+DHA	38.60 ± 1.93	33.89 ± 1.58	36.23 ± 0.70	34.29 ± 0.66
DHA/EPA^e^	2.40 ± 0.01^c^	1.94 ± 0.22^b^	1.22 ± 0.05^a^	1.02 ± 0.04^a^

The mean values of three replicates ± S.E.M. Difference of superscript letters in the same row indicate statistical significance (*P* < 0.05). ^a^SFA: saturated fatty acids; ^b^MUFA: mono-unsaturated fatty acids; ^c^n-6 PUFA: n-6 polyunsaturated fatty acids; ^d^n-3 PUFA: n-3 polyunsaturated fatty acids; ^e^DHA/EPA: the ratio of 22:6n-3 to 20:5n-3.

## Data Availability

The data used to support the findings of this study are included within the article.

## References

[1] Gatlin D. M., Barrows F. T., Brown P. (2007). Expanding the utilization of sustainable plant products in aquafeeds: a review. *Aquaculture Research*.

[2] Hardy R. W. (2010). Utilization of plant proteins in fish diets: effects of global demand and supplies of fishmeal. *Aquaculture Research*.

[3] Yao J. T., Gu D. T., Kong C., Ju M., Hua X. M. (2020). Effect of soybean antigenic protein on feed palatability of fishmeal replaced diets for obscure puffer (*Takifugu fasciatus*) and the alternation of diet preference by domestication. *Aquaculture Reports*.

[4] FAO (Food and Agriculture Organization of the United Nations) (2012). *The State of World Fisheries and Aquaculture*.

[5] National Research Council (NRC) (2011). *Nutrient Requirements of Fish and Shrimp*.

[6] Francis G., Makkar H. P., Becker K. (2001). Antinutritional factors present in plant-derived alternate fish feed ingredients and their effects in fish. *Aquaculture*.

[7] Xie S. W., Zhou W. W., Tian L. X., Niu J., Liu Y. J. (2016). Effect of N-acetyl cysteine and glycine supplementation on growthperformance, glutathione synthesis, anti-oxidative and immune ability of Nile tilapia, *Oreochromis niloticus*. *Fish & Shellfish Immunology*.

[8] Atkinson A., Siegel V., Pakhomov E. A., Jessopp M. J., Loeb V. (2009). A re-appraisal of the total biomass and annual production of Antarctic krill. *Deep Sea Research, Part I: Oceanographic Research Papers*.

[9] Saleh R., Burri L., Benitez-Santana T., Turkmen S., Castro P., Izquierdo M. (2018). Dietary krill meal inclusion contributes to better growth performance of gilthead seabream juveniles. *Aquaculture Research*.

[10] Olsen R., Suontama J., Langmyhr E. (2006). The replacement of fish meal with Antarctic krill, *Euphausia superba* in diets for Atlantic salmon, Salmo salar. *Aquaculture Nutrition*.

[11] Tibbetts S. M., Milley J. E., Lall S. P. (2006). Apparent protein and energy digestibility of common and alternative feed ingredients by Atlantic cod, *Gadus morhua* (Linnaeus, 1758). *Aquaculture*.

[12] Batetta B., Griinari M., Carta G. (2009). Endocannabinoids may mediate the ability of (n-3) fatty acids to reduce ectopic fat and inflammatory mediators in obese Zucker rats. *Journal of Nutrition*.

[13] Liu L., Bartke N., Dacle H. V. (2014). Higher efficacy of dietary DHA provided as a phospholipid than as a triglyceride for brain DHA accretion in neonatal piglets. *Journal of Lipid Research*.

[14] Lu F. S. H., Bruheim I., Ale M. T., Jacobsen C. (2015). The effect of thermal treatment on the quality changes of Antartic krill meal during the manufacturing process: high processing temperatures decrease product quality. *European Journal of Lipid Science and Technology*.

[15] Ambati R. R., Phang S. M., Ravi S., Aswathanarayana R. G. (2014). Astaxanthin: sources, extraction, stability, biological activities and its commercial applications-a review. *Marine Drugs*.

[16] Wade N. M., Gabaudan J., Glencross B. D. (2017). A review of carotenoid utilisation and function in crustacean aquaculture. *Reviews in Aquaculture*.

[17] Suontama J., Kiessling A., Melle W., Waagbo R., Olsen R. E. (2007). Protein from Northern krill (*Thysanoessa inermis*), Antarctic krill (*Euphausia superba*) and the Arctic amphipod (*Themisto libellula*) can partially replace fish meal in diets to Atlantic salmon (*Salmo salar*) without affecting product quality. *Aquaculture Nutrition*.

[18] Hatlen B., Berge K., Nordrum S., Johnsen K., Kolstad K., Mørkøre T. (2017). The effect of low inclusion levels of Antarctic krill (*Euphausia superba*) meal on growth performance, apparent digestibility and slaughter quality of Atlantic salmon (*Salmo salar*). *Aquaculture Nutrition*.

[19] Gao R. J., Chen L., Zhang W., Zhang S., Rao J., Hu J. (2020). Effect of dietary Antarctic krill *Euphausia superba* on the growth performance and nonspecifific immunity of red swamp crayfifish *Procambarus clarkia*. *Fish & Shellfish Immunology*.

[20] Yoshitomi B., Aoki M., Oshima S., Hata K. (2006). Evaluation of krill (*Euphausia superba*) meal as a partial replacement for fish meal in rainbow trout (*Oncorhynchus mykiss*) diets. *Aquaculture*.

[21] Yoshitomi B., Nagano I. (2012). Effect of dietary fluoride derived from Antarctic krill (*Euphausia superba*) meal on growth of yellowtail (*Seriola quinqueradiata*). *Chemosphere*.

[22] Lacson C. F. Z., Lu M. C., Huang Y. H. (2020). Fluoride network and circular economy as potential model for sustainable development-a review. *Chemosphere*.

[23] Wang Y. B., Ye T., Wang X. G., Zhou C. Y. (2017). Impact of main factors on the catch of Portunus trituberculatus in the Northern East China Sea. *Pakistan Journal of Zoology*.

[24] China Fishery Statistical Yearbook (2017). *Compiled by Fishery Bureau of China Agriculture Department*.

[25] Wang X. X., Yuan Y., Li C. C. (2020). Partial substitution of fish meal with soy protein concentrate in commercial diets for juvenile swimming crab, *Portunus trituberculatus*. *Animal Feed Science and Technology*.

[26] Xie S. C., Zhou Q. C., Zhang X. S. (2022). Effect of dietary replacement of fish meal with low-gossypol cottonseed protein concentrate on growth performance and expressions of genes related to protein metabolism for swimming crab (*Portunus trituberculatus*). *Aquaculture*.

[27] Jin M., Zhou Q. C., Zhang W., Xie F. J., ShenTu J. K., Huang X. L. (2013). Dietary protein requirements of the juvenile swimming crab, *Portunus trituberculatus*. *Aquaculture*.

[28] Yuan Y., Wang X. X., Jin M., Sun P., Zhou Q. C. (2019). Influence of different lipid sources on growth performance, oxidation resistance and fatty acid profiles of juvenile swimming crab, *Portunus trituberculatus*. *Aquaculture*.

[29] Aoac (1990). *Official Methods of Analysis*.

[30] Harris E. L. V. (1988). *Amino acid Analysis by Precolumn Derivatization*.

[31] Luo J. X., Zhu T. T., Wang X. X. (2020). Toxicological mechanism of excessive copper supplementation: effects on coloration, copper bioaccumulation and oxidation resistance in mud crab *Scylla paramamosain*. *Journal of Hazardous Materials*.

[32] Ding L. Y., Jin M., Sun P. (2017). Cloning, tissue expression of the fatty acid-binding protein (Pt-FABP1) gene, and effects of dietary phospholipid levels on fabp and vitellogenin gene expression in the female swimming crab *Portunus trituberculatus*. *Aquaculture*.

[33] Torrecillas S., Montero D., Carvalho M., Benitez-Santana T., Izquierdo M. (2021). Replacement of fish meal by Antarctic krill meal in diets for European sea bass *Dicentrarchus labrax*: growth performance, feed utilization and liver lipid metabolism. *Aquaculture*.

[34] Shi Y., Zhong L., Zhang J. Z. (2021). Substitution of fish meal with krill meal in rice field eel (*Monopterus albus*) diets: effects on growth, immunity, muscle textural quality, and expression of myogenic regulation factors. *Animal Feed Science and Technology*.

[35] Tharaka K., Benitez-Santana T., Gunathilaka B. E. (2020). Evaluation of Antarctic krill (*Euphausia superba*) meal supplementation in diets for olive flounder (*Paralichthys olivaceus*).

[36] Suresh A. V., Vasagam K. P. K., Nates S. (2011). Attractability and palatability of protein ingredients of aquatic and terrestrial animal origin, and their practical value for blue shrimp, *Litopenaeus stylirostris* fed diets formulated with high levels of poultry byproduct meal. *Aquaculture*.

[37] Derby C. D., Elsayed F. H., Williams S. A. (2016). Krill meal enhances performance of feed pellets through concentration-dependent prolongation of consumption by Pacific white shrimp, *Litopenaeus vannamei*. *Aquaculture*.

[38] Nunes A., Sa M., Sabry-Neto H. (2011). Growth performance of the white shrimp, *Litopenaeus vannamei*, fed on practical diets with increasing levels of the Antarctic krill meal, *Euphausia superba*, reared in clear- versus green-water culture tanks. *Aquaculture Nutrition*.

[39] Aksnes A., Hope B., Jönsson E., Björnsson B. T., Albrektsen S. (2006). Size-fractionated fish hydrolysate as feed ingredient for rainbow trout (*Oncorhynchus mykiss*) fed high plant protein diets. I: growth, growth regulation and feed utilization. *Aquaculture*.

[40] Thompson K. R., Muzinic L. A., Engler L. S., Webster C. D. (2005). Evaluation of practical diets containing different protein levels, with or without fish meal, for juvenile Australian red claw crayfish (*Cherax quadricarinatus*). *Aquaculture*.

[41] Albrektsen S., Mundheim H., Aksnes A. (2006). Growth, feed efficiency, digestibility and nutrient distribution in Atlantic cod (*Gadus morhua*) fed two different fish meal qualities at three dietary levels of vegetable protein sources. *Aquaculture*.

[42] Lim C., Ako H., Brown C. L., Hahn K. (1997). Growth response and fatty acid composition of juvenile *Penaeus vannamei* fed different sources of dietary lipid. *Aquaculture*.

[43] Zhou Q. C., Li C. C., Liu C. W., Chi S. Y., Yang Q. H. (2007). Effects of dietary lipid sources on growth and fatty acid composition of juvenile shrimp, *Litopenaeus vannamei*. *Aquaculture Nutrition*.

[44] Li J. Y., Guo Z. L., Gan X. H., Wang D. L., Zhang M. F., Zhao Y. L. (2011). Effect of different dietary lipid sources on growth and gonad maturation of pre-adult female *Cherax quadricarinatus* (von martens). *Aquaculture Nutrition*.

[45] Han T., Wang J. T., Hu S. X., Li X. Y., Jiang Y. D., Wang C. L. (2015). Effects of different dietary lipid sources on growth performance and tissue fatty acid composition of juvenile swimming crab *Portunus trituberculatus*. *Chinese Journal of Oceanology and Limnology*.

[46] Shang T. T., Liu L., Zhou J. (2017). Protective effects of various ratios of DHA/EPA supplementation on high-fat diet-induced liver damage in mice. *Lipids in Health and Disease*.

[47] Lv J. J., Liu P., Wang Y., Gao B. Q., Chen P., Li J. (2013). Transcriptome analysis of *Portunus trituberculatus* in response to salinity stress provides insights into the molecular basis of osmoregulation. *PLoS One*.

[48] Phaniendra A., Jestadi D. B., Periyasamy L. (2015). Free radicals: properties, sources, targets, and their implication in various diseases. *Indian Journal of Clinical Biochemistry*.

[49] Chatgilialoglu C., Ferreri C., Krokidis M. G., Masi A., Terzidis M. A. (2021). On the relevance of hydroxyl radical to purine DNA damage. *Free Radical Research*.

[50] He H. L., Chen X. L., Sun C. Y., Zhang Y. Z., Gao P. J. (2006). Preparation and functional evaluation of oligopeptide-enriched hydrolysate from shrimp (*Acetes chinensis*) treated with crude protease from Bacillus sp. SM98011. *Bioresource Technology*.

[51] Fakhri S., Abbaszadeh F., Dargahi L., Jorjani M. (2018). Astaxanthin: a mechanistic review on its biological activities and health benefits. *Pharmacological Research*.

[52] Han T., Li X. Y., Wang J. T., Wang C. L., Yang M., Zheng P. Q. (2018). Effects of dietary astaxanthin (AX) supplementation on pigmentation, antioxidant capacity and nutritional value of swimming crab, *Portunus trituberculatus*. *Aquaculture*.

[53] Ju Z. Y., Deng D. F., Dominy W. G., Forster L. P. (2011). Pigmentation of Pacific white shrimp, *Litopenaeus vannamei*, by dietary astaxanthin extracted from *Haematococcus pluvialis*. *Journal of the World Aquaculture Society*.

[54] Ali-Nehari A., Kim S. B., Lee Y. B., Lee H. Y., Chun B. S. (2012). Characterization of oil including astaxanthin extracted from krill (*Euphausia superba*) using supercritical carbon dioxide and organic solvent as comparative method. *Korean Journal of Chemical Engineering*.

[55] Laplante M., Sabatini D. M. (2012). mTOR signaling in growth control and disease. *Cell*.

[56] Mykles D. L. (2021). Signaling pathways that regulate the crustacean molting gland. *Frontiers in Endocrinology*.

[57] Covi J. A., Chang E. S., Mykles D. L. (2012). Neuropeptide signaling mechanisms incrustacean and insect molting gland. *Invertebrate Reproduction & Development*.

[58] Ma X., Blenis J. (2009). Molecular mechanisms of mTOR-mediated translational control. *Nature Reviews Molecular Cell Biology*.

[59] Magnuson B., Ekim B., Fingar D. C. (2012). Regulation and function of ribosomal protein S6 kinase (S6K) within mTOR signalling networks. *Biochemical Journal*.

[60] Cota D., Proulx K., Smith K. A. (2006). Hypothalamic mTOR signaling regulates food intake. *Science*.

[61] Yin B., Liu H. Y., Tan B. P. (2021). MHC II-PI3K/Akt/mTOR signaling pathway regulates intestinal immune response induced by soy glycinin in hybrid grouper: protective effects of sodium butyrate. *Frontiers in Immunology*.

[62] Teleman A. A. (2010). Molecular mechanisms of metabolic regulation by insulin in *Drosophila*. *Biochemical Journal*.

[63] Fang F. (2013). Antibiotic and ROS linkage questioned. *Nature Biotechnology*.

